# Weakly-supervised convolutional neural networks of renal tumor segmentation in abdominal CTA images

**DOI:** 10.1186/s12880-020-00435-w

**Published:** 2020-04-15

**Authors:** Guanyu Yang, Chuanxia Wang, Jian Yang, Yang Chen, Lijun Tang, Pengfei Shao, Jean-Louis Dillenseger, Huazhong Shu, Limin Luo

**Affiliations:** 1 LIST, Key Laboratory of Computer Network and Information Integration, Southeast University, Ministry of Education, Nanjing, China; 2 Centre de Recherche en Information Biomédicale Sino-Français (CRIBs), Rennes, France; 3 Beijing Engineering Research Center of Mixed Reality and Advanced Display, School of Optics and Electronics, Beijing Institute of Technology, Beijing, 100081 China; 4 Department of Radiology, The First Affiliated Hospital of Nanjing Medical University, Nanjing, China; 5 Department of Urology, The First Affiliated Hospital of Nanjing Medical University, Nanjing, China; 6 University Rennes, Inserm, LTSI - UMR1099, F-35000 Rennes, France

**Keywords:** Weakly-supervised, Renal tumor segmentation, Bounding box, Convolutional neural network

## Abstract

**Background:**

Renal cancer is one of the 10 most common cancers in human beings. The laparoscopic partial nephrectomy (LPN) is an effective way to treat renal cancer. Localization and delineation of the renal tumor from pre-operative CT Angiography (CTA) is an important step for LPN surgery planning. Recently, with the development of the technique of deep learning, deep neural networks can be trained to provide accurate pixel-wise renal tumor segmentation in CTA images. However, constructing the training dataset with a large amount of pixel-wise annotations is a time-consuming task for the radiologists. Therefore, weakly-supervised approaches attract more interest in research.

**Methods:**

In this paper, we proposed a novel weakly-supervised convolutional neural network (CNN) for renal tumor segmentation. A three-stage framework was introduced to train the CNN with the weak annotations of renal tumors, i.e. the bounding boxes of renal tumors. The framework includes pseudo masks generation, group and weighted training phases. Clinical abdominal CT angiographic images of 200 patients were applied to perform the evaluation.

**Results:**

Extensive experimental results show that the proposed method achieves a higher dice coefficient (DSC) of 0.826 than the other two existing weakly-supervised deep neural networks. Furthermore, the segmentation performance is close to the fully supervised deep CNN.

**Conclusions:**

The proposed strategy improves not only the efficiency of network training but also the precision of the segmentation.

## Background

Renal cancer is one of the ten most common cancers in human beings. The minimally invasive laparoscopic partial nephrectomy (LPN) is now increasingly used to treat the renal cancer [1]. In the clinical practice, some anatomical information such as the location and the size of the renal tumor is very important for the LPN surgery planning. However, manual delineation of the contours of the renal tumor and kidney in the pre-operative CT images including more than 200 slices is a time-consuming work. In recent years, deep neural networks have been the widely used for organ and lesion segmentation in medical images [2]. However, fully-supervised deep neural networks were trained by a large number of training images with pixel-wise labels, which take a considerable time for radiologists to build. Thus, weakly supervised approaches attract more interest, especially for medical image segmentation.

In recent years, several weakly-supervised CNNs have been developed for semantic segmentation in natural images. According to the weak annotations used for CNN training, these approaches can be divided into four main categories: bounding box [3–6], scribble [7, 8], points [9, 10] and image-level labels [11–17]. However, as far as we know, there are only a few weakly-supervised methods reported for the segmentation tasks in medical images. DeepCut [18] adopted an iterative optimization method to train CNNs for brain and lung segmentation with the bounding-box labels which are determined by two corner coordinates, and the target object is inside the bounding box. In another weakly-supervised scenario [19], fetal brain MR images were segmented using a fully convolutional network (FCN) trained by super-pixel annotations [20] which refer to an irregular region composed of adjacent pixels with similar texture, color, brightness or other features. Kervadec et al. [21] conducted a size loss on CNN, which was used to obtain the segmentation of different organs from the scribbled annotations which annotate different areas and their classes. These weakly learned-based methods have achieved comparable accuracy on normal organs but have not yet been applied to lesions. The approaches for renal tumor segmentation are mainly based on traditional methods such as level-set [22], SVM [23] and fully-supervised deep neural networks [24, 25]. To the best of our knowledge, there is no weakly-supervised deep learning technique reported for renal tumor segmentation.

**Fig. 1:**
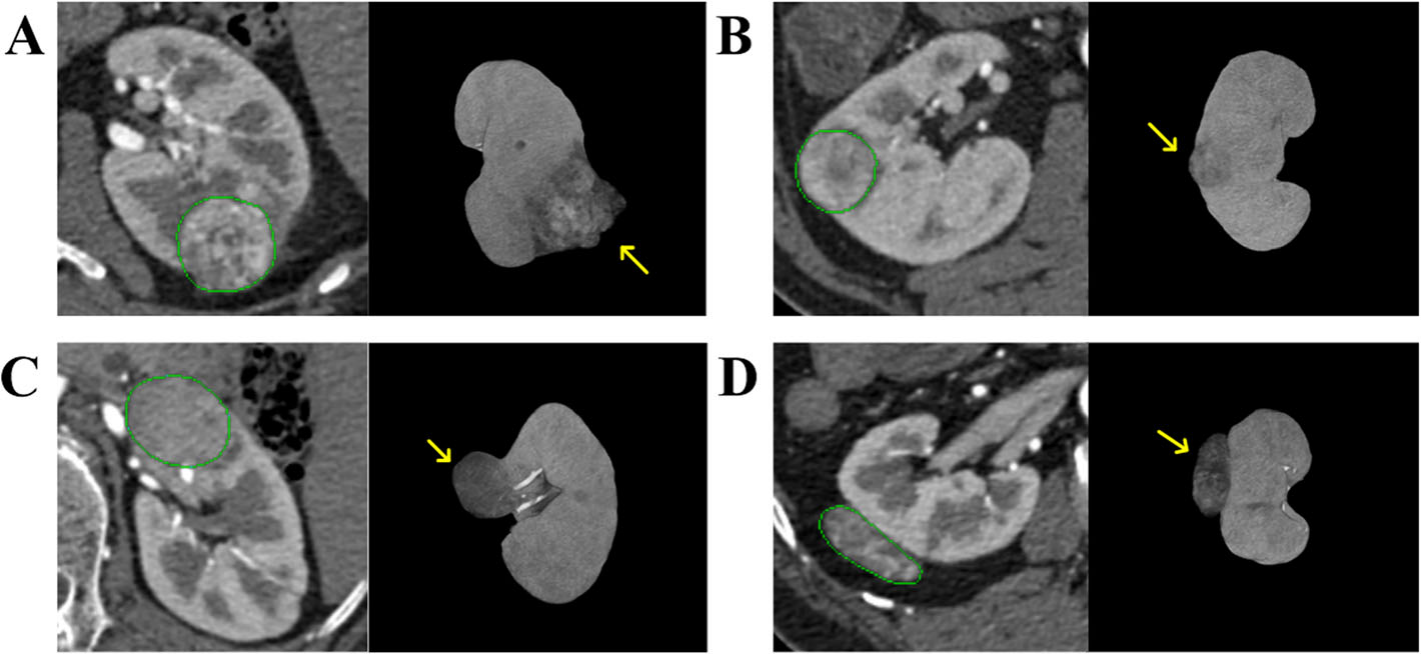
Four contrast-enhanced CT images of different pathological renal tumors. The tumors are marked by yellow arrows in 3D views. The manual contours of the renal tumors delineated by a radiologist are displayed in 2D slices. The pathological subtypes of the renal tumors are clear cell renal cell carcinoma (RCC) in (**a**) and (**b**), chromophobe RCC in (**c**) and angiomyolipoma in (**d**)

As shown in Fig. 1, the precise segmentation of renal tumors is a challenging task because of the large variation of the size, location, intensity and image texture of renal tumors in CTA images. For example, small tumors are often overlooked since they are difficult to be distinguished from the normal tissue, as displayed in Fig. 1(b). Different pathological types of renal tumors show varied intensities and textures which increases the difficulty of segmentation [26]. Thus, the segmentation of renal tumors by a weakly-supervised method is still an open problem.

In this paper, bounding boxes of renal tumors are provided as weak annotations to train a CNN which can generate pixel-wise segmentation of renal tumors. Compared to the other types of annotations, the bounding box is a simple way to be defined by radiologists [27]. The main contributions of this paper are as follows:

1. To the best of our knowledge, we proposed a weakly-supervised CNN for renal tumor segmentation for the first time.
2. The proposed method can accomplish network training faster and overcome the under-segmentation problem compared with the iterative training strategy usually adopted by the other weakly-supervised CNNs [18, 28].
3. The experimental results of a 200-patients clinical dataset with different pathological types of renal tumors show that the CNN trained by our method can provide precise renal tumor segmentation.

The remaining paper is organized as follows: Materials section describes the datasets used in this paper. In Methods section the method is introduced in detail. Experimental results are summarized in Results section. We give extra discussion in Discussion section, a conclusion in Conclusion section and abbreviations section. The last section is the declarations of this paper.

## Materials

The pre-operative CT images of 200 patients who underwent an LPN surgery were included in this study. The CT images were generated on a Siemens dual-source 64-slice CT scanner. The contrast media was injected during the CT image acquisition. The study was already approved by the institutional review board of Nanjing Medical University. Two scan phases including arterial and excretion phases were performed for data acquisition. In this paper, CT images acquired in arterial phase were used for training and testing. The arterial scan was triggered by the bolus tracking technique after 100 ml of contrast injection (Ultravist 370, Schering) in the antecubital vein at a velocity of 5 ml/s. Bolus tracking used for timing and scanning was started automatically 6 s after contrast enhancement reached 250HU in a region of interest (ROI) placed in the descending aorta. The pixel size of these CT images is between 0.56mm^2^ to 0.74mm^2^. The slice thickness and the spacing in z-direction were fixed at 0.75 mm and 0.5 mm respectively. After LPN surgery, pathological tests were performed to examine the pathological types of renal tumors. Five types of renal tumors were included in this study, i.e. clear cell RCC (172 patients), chromophobe RCC (4 patients), papillary RCC (6 patients), oncocytoma (6 patients) and angiomyolipoma (12 patients). The volume of the renal tumors’ ranges from 12.21 ml to 159.67 ml and the mean volume is 42.58 ml.

**Fig. 2:**
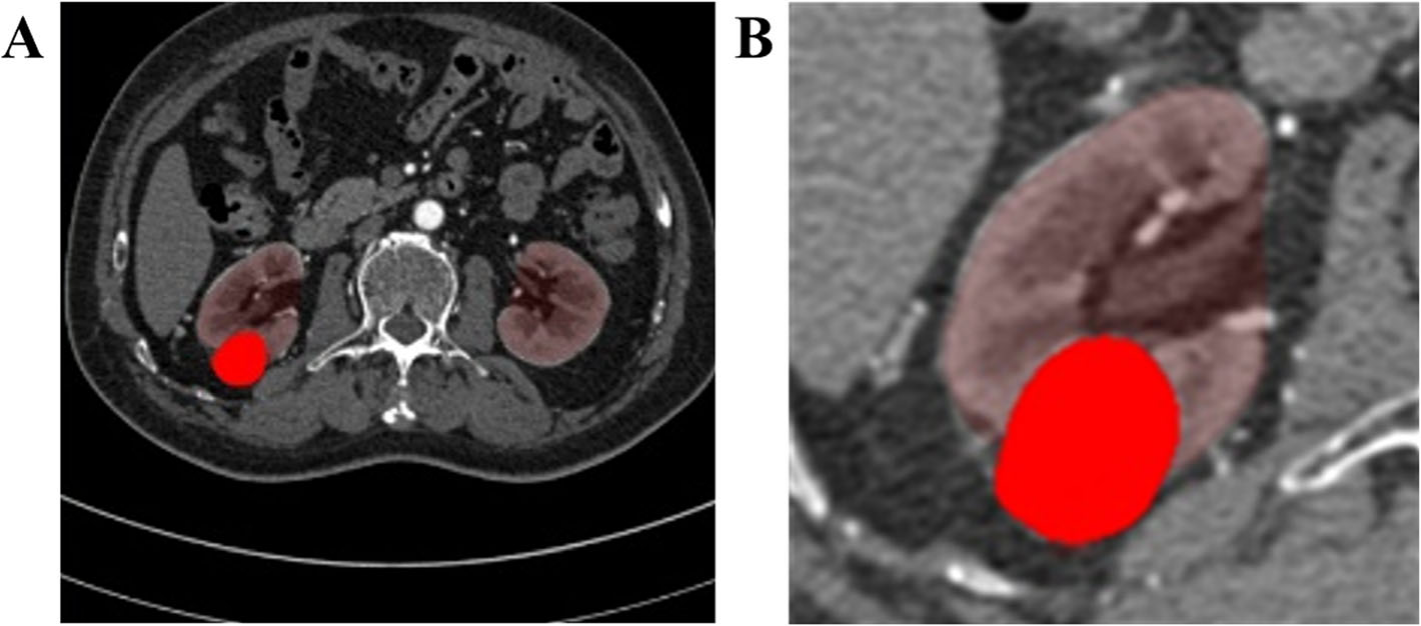
**a** The original image with labeled kidney and renal tumor. The region in red represents renal tumor. **b** The cropped original image with the label for renal tumor segmentation

As shown in Fig. 2(a), each original CT image was resampled to an isotropic volume with the size of axial slice equal to 512*512. The original CT image contained the entire abdomen, whereas only the area of the kidney needed to be considered in this experiment. Thus, the kidneys in the images were firstly segmented by the multi-atlas-based method [29] to define the ROIs of kidneys as shown in Fig. 2(b). The multi-atlas-based method just produce initial segmentation of kidneys, two radiologists checked the contours of kidneys and corrected them if necessary. The contours of tumors were drawn manually by one radiologist with 7-years’ experience and checked by another radiologist with 15-years’ experience in the cross-sectional slices. However, the pixel-wise masks were only used for bounding boxes generation and testing dataset evaluation. Among 200-patient images, 120 patients were selected to build the training dataset and the other 80 patients were used as the testing dataset.

## Methods

We train our proposed method via bounding boxes of renal tumors to obtain pixel-wise segmentation. Thus, a pre-processing step is performed before the training procedure of weakly-supervised model. In Pre-processing section, the pre-processing including normalization and bounding box generation is briefly introduced. Then the proposed weakly-supervised method is illustrated in detail in Weakly supervised segmentation from bounding box Section. Finally, the parameters of training are explained in Training section.

### Pre-processing

#### Normalization

As is done in other studies, original CT images should be normalized before fed into the neural network. Due to the existence of bones, contrast media and air in the intestinal tract, CT values in the abdominal CT image or extracted ROIs can range from -1000HU to more than 800HU. Thus, Hounsfield values were clipped to a range of − 200 to 500 HU. After thresholding, the pixel values in all images are normalized to 0~1 by Min-Max Normalization:

1 $$ {X}^{\prime }=\frac{X-{X}_{min}}{X_{max}-{X}_{min}} $$

#### Bounding box generation

**Fig. 3:**
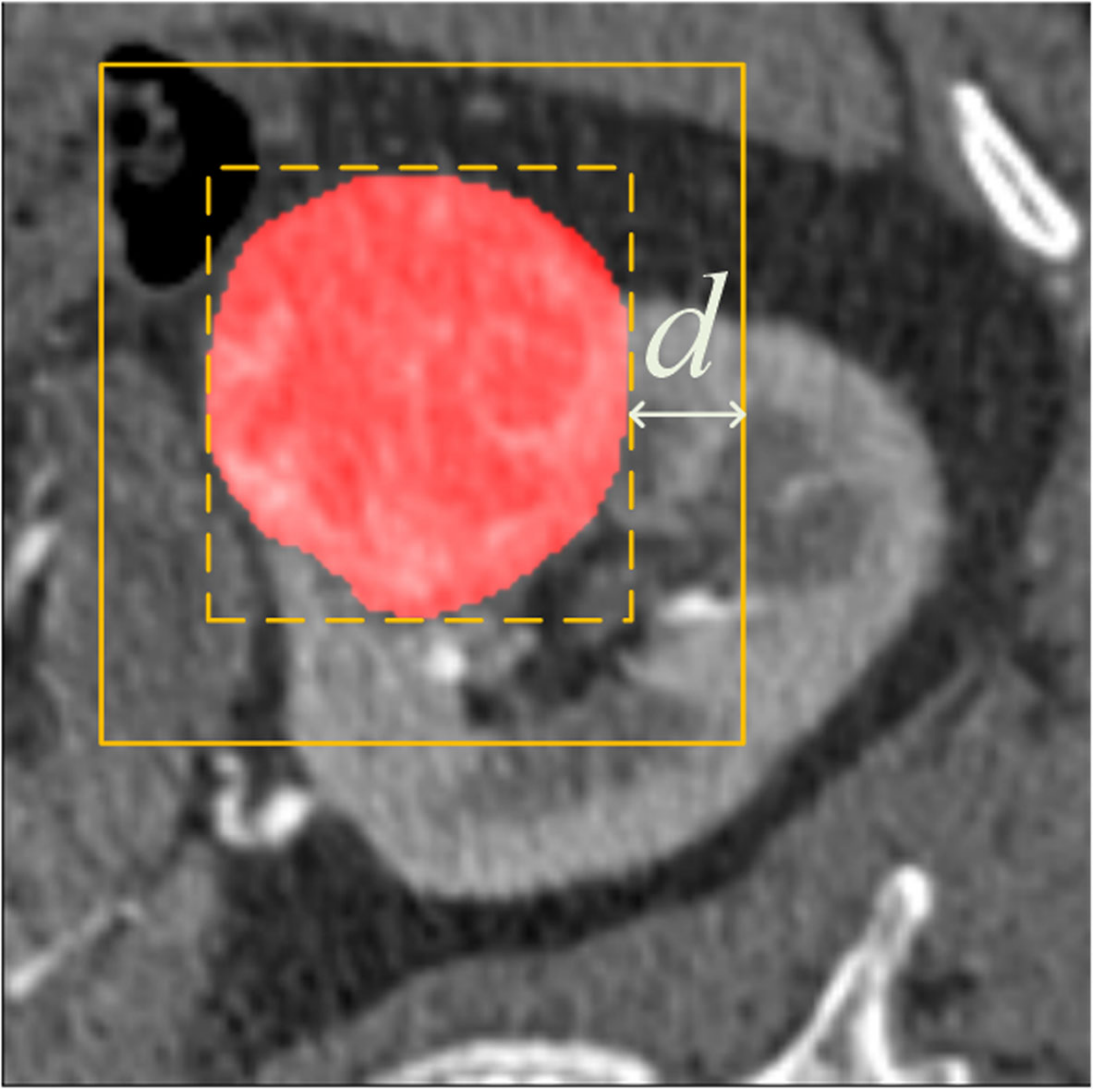
The bounding box with margin *d* is defined as weak annotations according to the label of renal tumors

In this paper, bounding boxes are generated by ground truth of renal tumors. As shown in Fig. 3, the bounding box of ground truth is shown in the dotted line. The parameter *d* in pixel represents the margin added to the bounding box in our experiment to generate different types of weak annotations. In addition, the reference labels of renal tumors in the training dataset were only used to generate bounding boxes and not used for CNN training, and the reference labels in the testing dataset were used for quantitative evaluation.

**Fig. 4:**
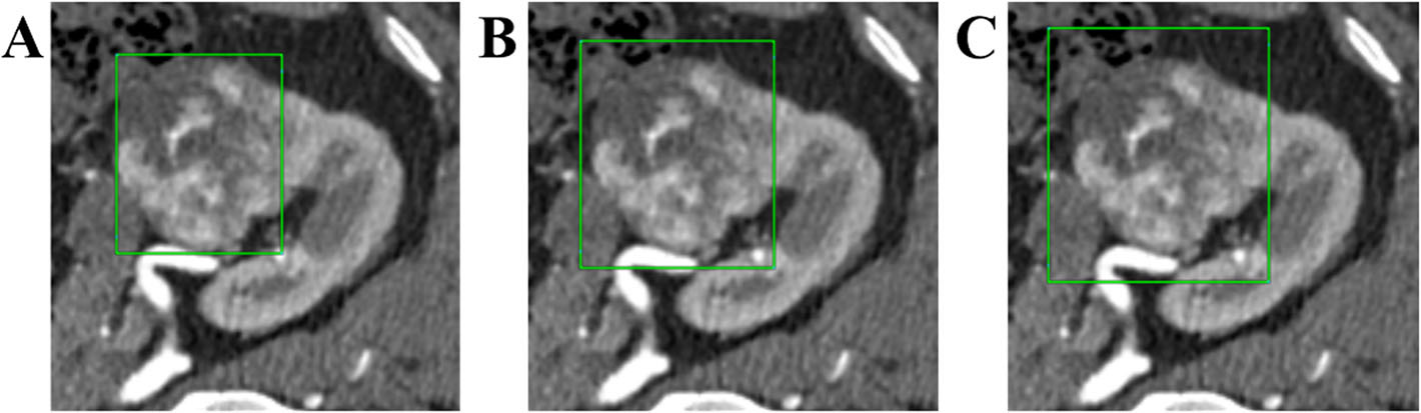
Comparison of bounding boxes with different margins. The 2D image is the maximum slice. Contours in green correspond to bounding boxes

The bounding boxes with different margins are defined according to the ground truth and used as weak annotations for CNN training. We set *d* to be 0, 5 and 10 pixels (Fig. 4(a)-(c)) in our study to simulate the manual weak annotations by radiologists. If the bounding boxes with margin *d* are beyond the range of images, it will be limited in the region of images. As shown in Fig. 4, the comparison of bounding boxes with different margin values is given.

### Weakly supervised segmentation from bounding box

**Fig. 5:**
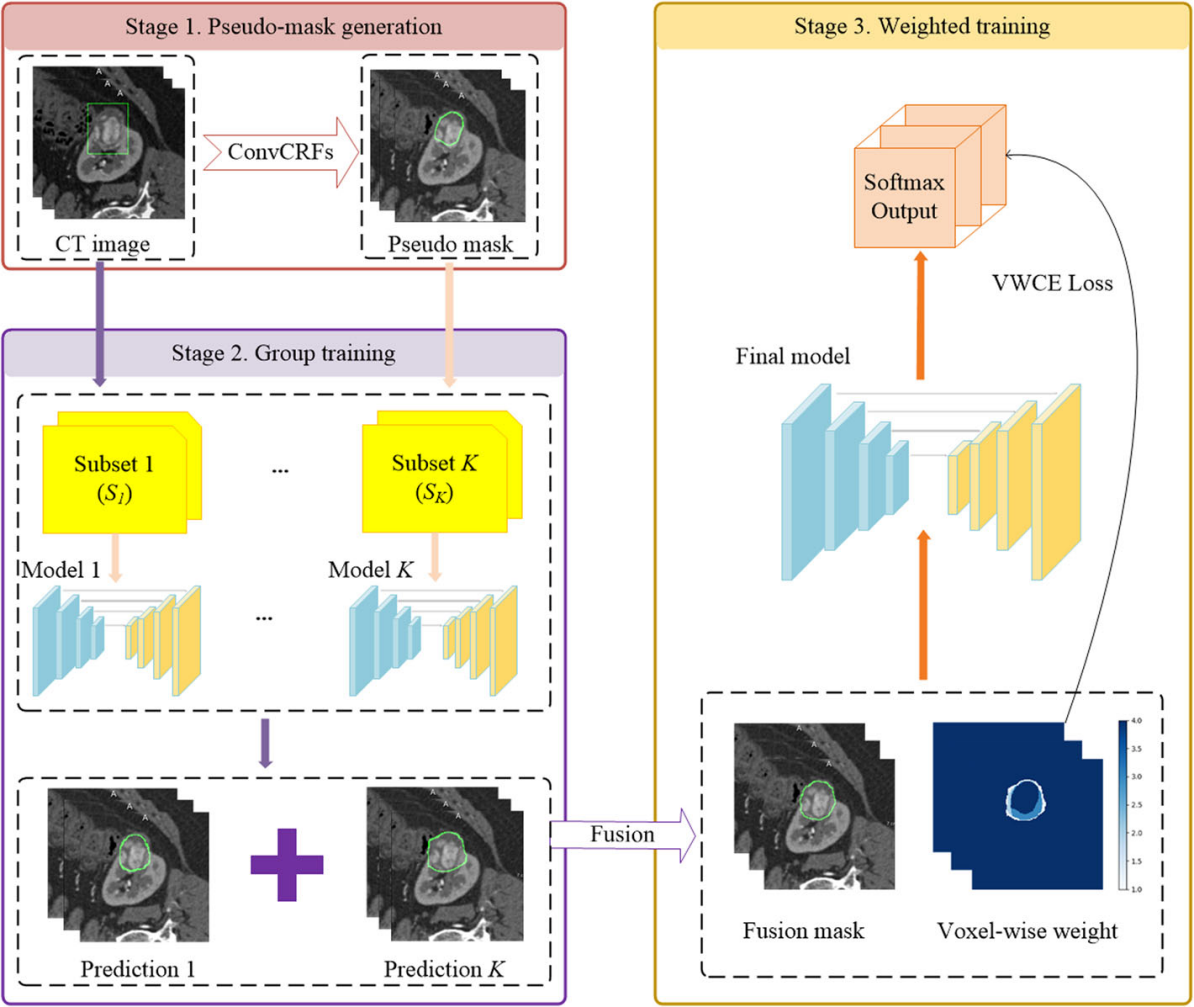
An overview of the proposed weakly-supervised method

Three main steps are included in the proposed method as shown in Fig. 5. Firstly, we get pseudo masks from bounding boxes by convolutional conditional random fields (ConvCRFs) [30]. Then, in the group training stage, several CNNs are trained by using pseudo masks. Fusion masks and voxel-wise weight map are generated based on the predictions of the CNNs trained in this stage. In the last stage of weighted training, the final CNN is trained by fusion masks and voxel-wise weighted cross-entropy (VWCE) loss function. These three main stages are described in the following Pseudo masks generation, Group training and fusion mask generation and Training with VWCE loss sections respectively.

#### Pseudo masks generation

As adopted by other methods [3, 18], the pseudo masks of renal tumors are generated from bounding boxes as initialization for CNN model training. The quality of pseudo masks influences the performance of CNN. Inspired by fully connected conditional random fields (CRFs) [31], this problem can be regarded as maximum a posteriori (MAP) inference in a CRF defined over pixels [5]. The CRF potentials take advantage of the context between pixels and encourage consistency between similar pixels. Suppose an image *X* = {*x*_1_…*x*_*N*_} and corresponding voxel-wise label *Y* = {*y*_1_…*y*_*N*_}, here *y*_*i*_ ∈ {0, 1}. *y*_*i*_ = 0 means *x*_*i*_ is located outside the bounding box, while *y*_*i*_ = 1 means *x*_*i*_ is located inside the bounding box. The CRF conforms to the Gibbs distribution. Then, the Gibbs energy can be defined as:

2 $$ E(X)={\sum}_iU\left({y}_i\right)+{\sum}_{i,j}P\left({y}_i,{y}_j\right) $$

where the first term is unary potential, representing the energy of assigning class *y*_*i*_ to the pixel *x*_*i*_, which is given by the bounding box. The latter term represents the pairwise potential, which is used to represent the energy of two pixels *x*_*i*_ and *x*_*j*_ in the image whose label are assigned to *y*_*i*_ and *y*_*j*_ respectively. In the fully connected CRFs, the pairwise potential function is defined as follows:

3 $$ P\left({y}_i,{y}_j\right)=\mu \left({y}_i,{y}_j\right){\sum}_{i\ne j\le N}w\bullet g\left({f}_i,{f}_j\right) $$

where *w* is a learnable parameter, *g* is the gaussian kernel defined by feature vectors *f* and *μ* is a label compatibility function.

However, because the volumetric image was used in our study, the computation of fully connected CRFs has high time complexity. Thus, inspired by Teichmann et al. [30], ConvCRFs were used for our pseudo masks generation. ConvCRFs adds the assumption of conditional independence into fully connected CRFs. Here, the matrix of gaussian kernel changes to:

4 $$ g\left({f}_i,{f}_j\right)=\mathit{\exp}\left(-{\sum}_{i\ne j\le D}\frac{f_i-{f}_j}{2{\theta}^2}\right) $$

where *θ* is a learnable parameter and *D* is the Manhattan distance between pixels *x*_*i*_ and *x*_*j*_, the pairwise energy is zero when the Manhattan distance exceeds *D*. The complexity of pairwise potential is simplified when conditional independence is added.

The merged kernel matrix *G* is calculated by ∑*w* · *g*, and the inference result is ∑*G* ∙ *X* which is similar to convolutions of CNNs. This assumption makes it possible to reformulate the inference in terms of convolutions in CRF, which can carry out efficient GPU calculation and complete feature learning. Thus, we can quickly get pseudo masks of renal tumors by minimizing the object function defined by Eq. (2).

#### Group training and fusion mask generation

Once we have generated pseudo masks of renal tumors, these masks are fed into CNN as weak labels for parameter learning. Most of weakly supervised segmentation methods used iterative training [5, 7] to optimize the accuracy of the weak labels from coarse to fine. However, the preliminary results showed that this iterative strategy is hard to improve the accuracy of pseudo masks due to the difficulties of the renal tumor segmentation mentioned before. To overcome this problem, we proposed a new CNN training strategy instead of iterative training method.

In the group training stage, we have input images {*X*_1_…*X*_*M*_} and pseudo masks {*I*_1_…*I*_*M*_}. The input training dataset is divided into *K* subsets {*S*_1_…*S*_*K*_}. For each subset *S*_*k*_, a CNN *f*(*X*; *θ*_*k*_), *X* ∈ *S*_*k*_ with parameter *θ*_*k*_ is trained. In total, we can get *K* CNNs trained in this stage. After that, for each image *X*_*m*_, we can get *K* predictions $\left\{{P}_m^1\dots {P}_m^K\right\}$ of renal tumors by these CNN models. We denote that ${P}_m^k=f\left({X}_m;{\theta}_k\right\}$. Pseudo code of group training is shown in Algorithm 1.

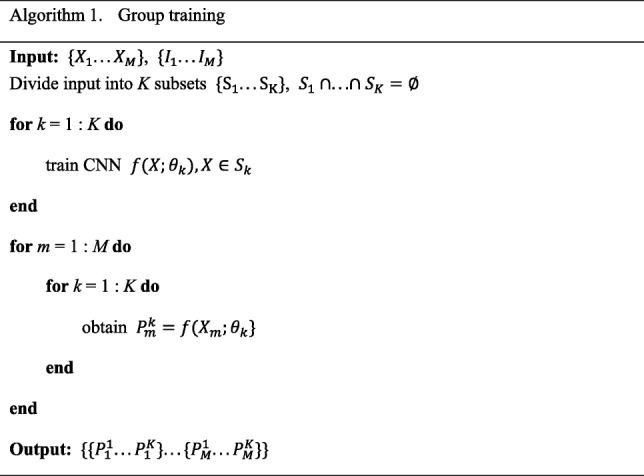



One thing worth to be mentioned is that one image in the training dataset is used to train only one CNN model in this stage. Once *K* CNN models are trained successfully, all the images in the training dataset will be used to test each CNN model and obtain *K* results for prediction. Thus, the proposed group training strategy can ameliorate the overfitting of the model. In order to alleviate the under-segmentation in the *K* predictions, a mask image is generated by fusing these predictions. The fusion mask is defined as follows:

5 $$ F{M}_m= ConvCRFs\left(P{M}_m\cup {P}_m^1\cup \dots \cup {P}_m^K\right) $$

where *FM* indicates the fusion masks, and *PM* indicates pseudo masks generated in Pseudo masks generation section. The ConvCRFs is adopted to refine the union of all prediction masks. The outputs of ConvCRFs will be used as the new weak labels for the next weighted training stage. In addition, a weight map is generated simultaneously which is defined as follows:

6 $$ {v}_m=P{M}_m+{P}_m^1+\dots +{P}_m^K,v\left[v=0\right]=K+1 $$

When the predicted label of a voxel is renal tumor in one prediction result, its *v*_*m*_ will be an integer within the range of 1 to *K* + 1. When *v*_*m*_ is equal to 0, its value will be reset to *K* + 1 to represent the weight of background.

#### Training with VWCE loss

After Section Pseudo masks generation and Group training and fusion mask generation, the fusion masks of training dataset are generated for the final CNN model training in this stage. Only the final CNN model will be used for testing dataset evaluation. In this stage, we train the CNN on the whole training dataset with the fusion masks. In addition, a new voxel-wise weighted cross-entropy (VWCE) loss function is designed to constrain the CNN training procedure. The traditional cross-entropy loss is defined as follows:

7 $$ {L}_{CE}=-\frac{1}{M}{\sum}_{m\in M}{\sum}_{c\in C}F{M}_{m,c}\mathit{\log}f\left({X}_{m,c};\theta \right) $$

where *FM* are fusion masks defined in Eq. (5), *f*(*X*; *θ*) are the outputs of CNN, *M* represents the number of samples and *C* represents the number of classes. In Eq. (7), pixels belonging to different classes have equal weight. In the case of unbalanced datasets, [32] proposed weighted cross-entropy loss defined as follows:

8 $$ {L}_{WCE}=-\frac{1}{M}{\sum}_{m\in M}{\sum}_{c\in C}{w}_cF{M}_{m,c}\mathit{\log}f\left({X}_{m,c};\uptheta \right) $$

where, *w*_*c*_ represents the weight of class *c*. Considering the weak annotations used in the training procedure, the voxel-wise weight map generated in the previous stage represents the probability of the predicted class given in the fusion mask. Thus, the voxel-wise weights obtained in Eq. (6) are introduced into Eq. (8) which is defined as follows:

9 $$ {L}_{VWCE}=-\frac{1}{M}{\sum}_{m\in M}{v}_m{\sum}_{c\in C}{w}_cF{M}_{m,c}\mathit{\log}f\left({X}_{m,c};\theta \right) $$

Finally, we conduct the final CNN model training with VWCE loss function on fusion masks. Our evaluations are all conducted on CNN trained in this stage.

### Training

#### Data augmentation

The ROIs of the pathological kidneys were cropped from the original images. The size of ROI is fixed at 150*150*N. Due to limited memory of GPU, the original ROIs were resampled to 128*128*64 before fed into the network. For each data, random crops and flipping were used for data augmentation. After data augmentation, the original 120 CT images were augmented into 14,400 images for the CNN training.

#### Parameter settings

The input are ROIs of kidneys and bounding boxes without any other annotations. Considering that UNet [32] has been widely used for medical image segmentation, we adopted UNet to be the CNN models in stage2 and stage3 in our experiments. The network parameters are updated by means of the back-propagation algorithm using the Adam optimizer. The initial learning rate was set to be 0.001 and decreased by $decay ed\_ learning\_ rate= learning\_ rate\ast decay\_{rate}^{\frac{global\_ step}{decay\_ step s}}$. In each epoch of training, it takes 3600 iterations to traverse all the training images with the batch size of 4. The class weights of cross-entropy *w*_c_ in Eqs. (8) and (9) were set to 1.0 and 0.2 for renal tumor and background respectively.

In stage2, we set the number of subset *K* to 3 for the training dataset of 120 CT images. Each subset contains 40 CT images. Three CNN models were trained to generate corresponding predictions of each training image. And fusion masks were generated by these predictions. The loss used in this stage is WCE loss defined in Eq. (8).

In stage3, the final CNN is trained by fusion masks as weak annotation labels. We evaluated the performance of the final CNN model with 80 patient images. In order to remove some misclassified outlier voxels, a connected component analysis with an 18-connectivity in 3D was carried out finally. The largest connected component in the output of the final CNN model was extracted as the segmentation results of renal tumors.

### Existing methods

We mainly compared with two weakly-supervised methods, i.e., SDI [5] and constrained-CNN [21]. The SDI method used 2D UNet to generate weak labels from bounding box by recursive training and carry out final segmentation. The weakly-supervised information used in the constrained-CNN method includes scribbles and the volume of target tissue. In this paper, the scribbles annotations used in constrained-CNN were generated by employing binary erosion on ground truth for every slice. Furthermore, the volumetric threshold of renal tumor was used in the loss function of Constrained-CNN. It was set to [0.9 *V*, 1.1 *V*], where *V* represents the volume of renal tumor in ground truth. As the architecture of UNet was used in [5, 21], as well as our proposed method, the UNet was trained by all the training dataset with the pixel-wise labels to generate a fully-supervised UNet model for extensive comparison.

## Results

Our method has been implemented using PyTorch framework in version 1.1.0. The network training and testing experiments were performed on a workstation with: CPU of i7-5930K, 128GB RAM and a GPU card of NVIDIA TITAN Xp of 12GB memory.

### The comparison of different weak labels and training losses

As shown in Table 1, DSCs between the different masks and the ground truth of the training dataset are displayed. The DSCs of bounding boxes are 0.666, 0.466 and 0.341 respectively when the margins of bounding box were set to 0, 5 and 10 pixels. The DSCs of pseudo masks generated by ConvCRFs can reach 0.862, 0.801 and 0.679. However, the DSCs of fusion masks generated after group training has even higher DSC than pseudo masks. Obviously, the rectangular bounding boxes were improved significantly by the Stage 1 and Stage 2.

**Table 1:** DSCs between different weak labels and ground truths of the training dataset

	Bounding boxes	Pseudo masks	Fusion masks
*d* = 0	0.666	0.862	**0.874**
*d* = 5	0.466	0.801	**0.810**
*d* = 10	0.341	0.679	**0.691**

**Fig. 6:**
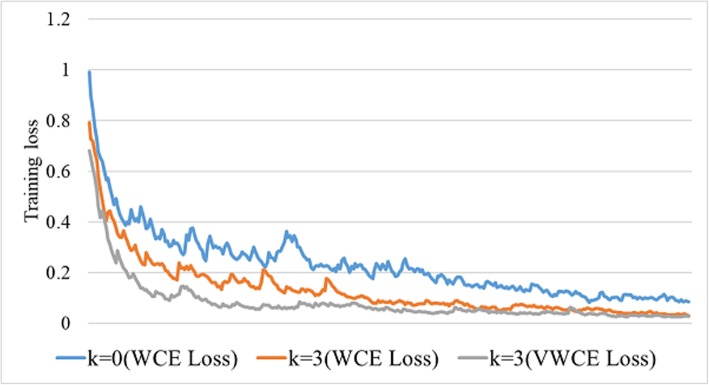
Training losses of the final CNN model in stage3 with different parameters

Furthermore, the improvements of the weak labels contribute to the training of the final CNN model. Figure 6 shows the training loss of the final CNN model with different parameters. Without group training, the training loss shows the slowest rate and the highest loss value during training. Contrarily, the usage of group training and VWCE loss makes the model converges faster and better.

### Evaluation of segmentation results of renal tumors in the testing dataset with different parameters

The DSC, Hausdorff distance (HD) [33] and average surface distance (ASD) were adopted to evaluate the segmentation results of our proposed method. The segmentation results of renal tumors in the testing dataset were obtained with different settings of parameters, i.e. number of groups, loss function and margin of bounding box. The comparison of DSCs in the testing dataset is displayed in Table 2. *k* = 0 means that the procedure of stage2 not used. In this situation, the pseudo masks generated by ConvCRFs were used as weak labels directly for the final CNN model training in the stage3. The loss functions used during the final model training is marked in the parentheses. MC represents the connected component analysis in the post-processing step.

**Table 2:** Comparison of segmentation results of testing dataset with different margins

		DSC	HD	ASD
*d* = 0	*k* = 0 (WCE Loss)	0.788	65.806	6.265
	*k* = 3 (WCE Loss)	0.822	34.187	3.889
	*k* = 3 (VWCE Loss)	0.834	40.617	3.361
	*k* = 3 (VWCE Loss) + 3D MC	**0.834**	**14.346**	**2.664**
*d* = 5	*k* = 0 (WCE Loss)	0.733	32.459	5.332
	*k* = 3 (WCE Loss)	0.784	70.948	7.988
	*k* = 3 (VWCE Loss)	0.820	37.633	3.879
	*k* = 3 (VWCE Loss) + 3D MC	**0.826**	**15.811**	**2.838**
*d* = 10	*k* = 0 (WCE Loss)	0.695	58.286	7.499
	*k* = 3 (WCE Loss)	0.720	81.611	7.804
	*k* = 3 (VWCE Loss)	0.741	36.127	4.672
	*k* = 3 (VWCE Loss) + 3D MC	**0.742**	**21.233**	**4.350**

#### The impact of group training

According to the values in Table 2, group training can effectively improve the DSC. The DSCs increased by 3.4, 5.1 and 2.5% when the margin of bounding box was set to 0, 5 and 10 pixels respectively.

#### The impact of VWCE loss

The usage of VWCE loss made further improvement of the DSC. The DSCs increased by 1.2, 3.6, and 2.1% respectively when the margin of bounding box was set to 0, 5 and 10 pixels. In addition, the application of VWCE loss and MC can alleviate the outliers in the segmentation result. The values of HD and ASD decreased significantly. Finally, the highest DSCs of 0.834, 0.826 and 0.742 can be achieved respectively when different margins of bounding box were set.

**Fig. 7:**
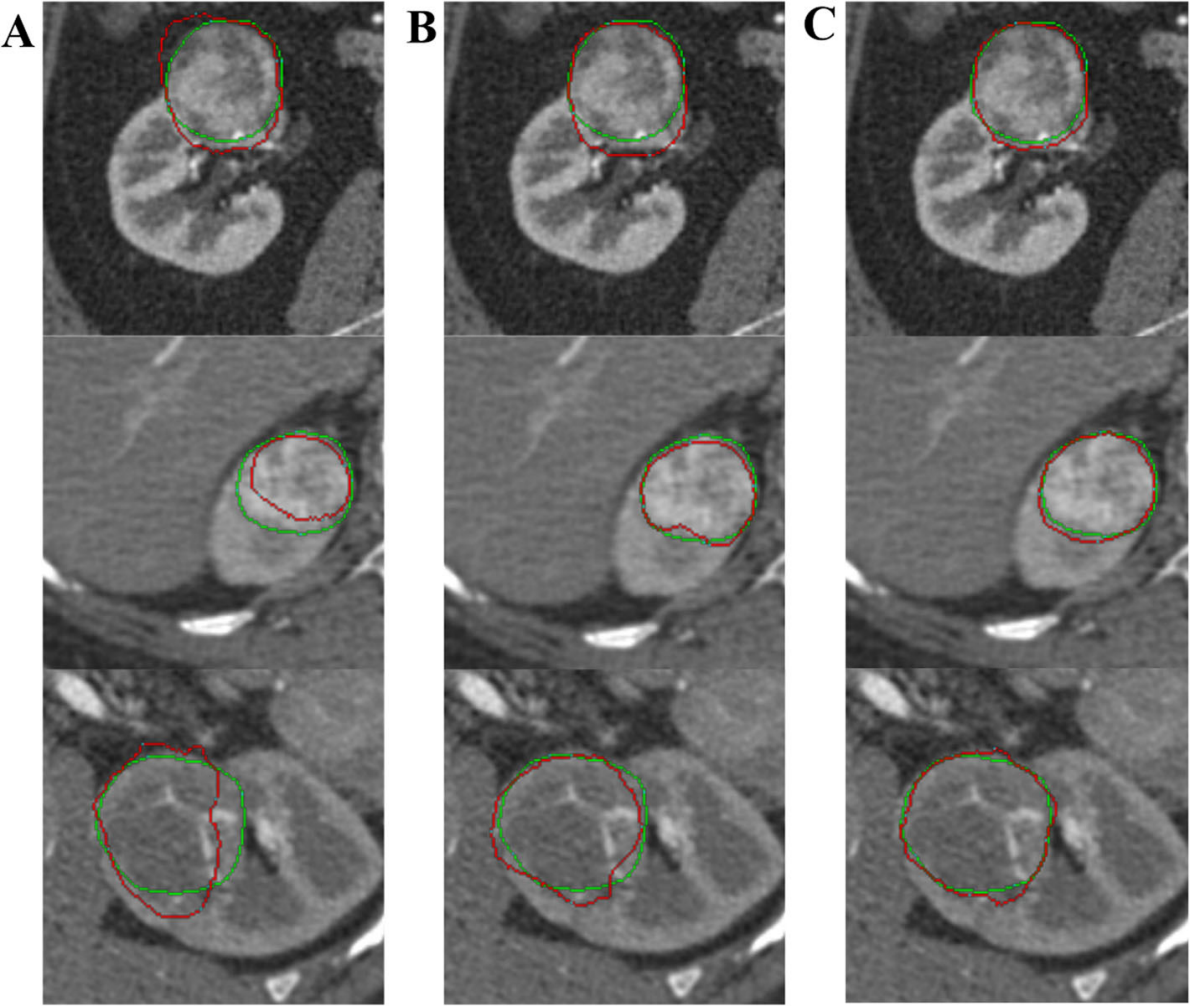
The comparison of 2D segmentation results with different parameters: *k* = 0 with WCE loss (**a**), *k* = 3 with WCE loss (**b**), *k* = 3 with VWCE loss (**c**). Contours in green and red correspond to ground truths and segmentation results respectively

Figure 7 Shows the 2D visualization of segmentation results with different parameters. Obviously, renal tumors cannot be segmented precisely without group training as shown in Fig. 7(a). With the application of group training, the over- or under-segmentation of tumors is significantly improved (Fig. 7b). However, the segmentations of the boundary are still imprecise. With the application of group training and VWCE loss function, the best segmentation results have been obtained as shown in Fig. 7(c)

**Fig. 8:**
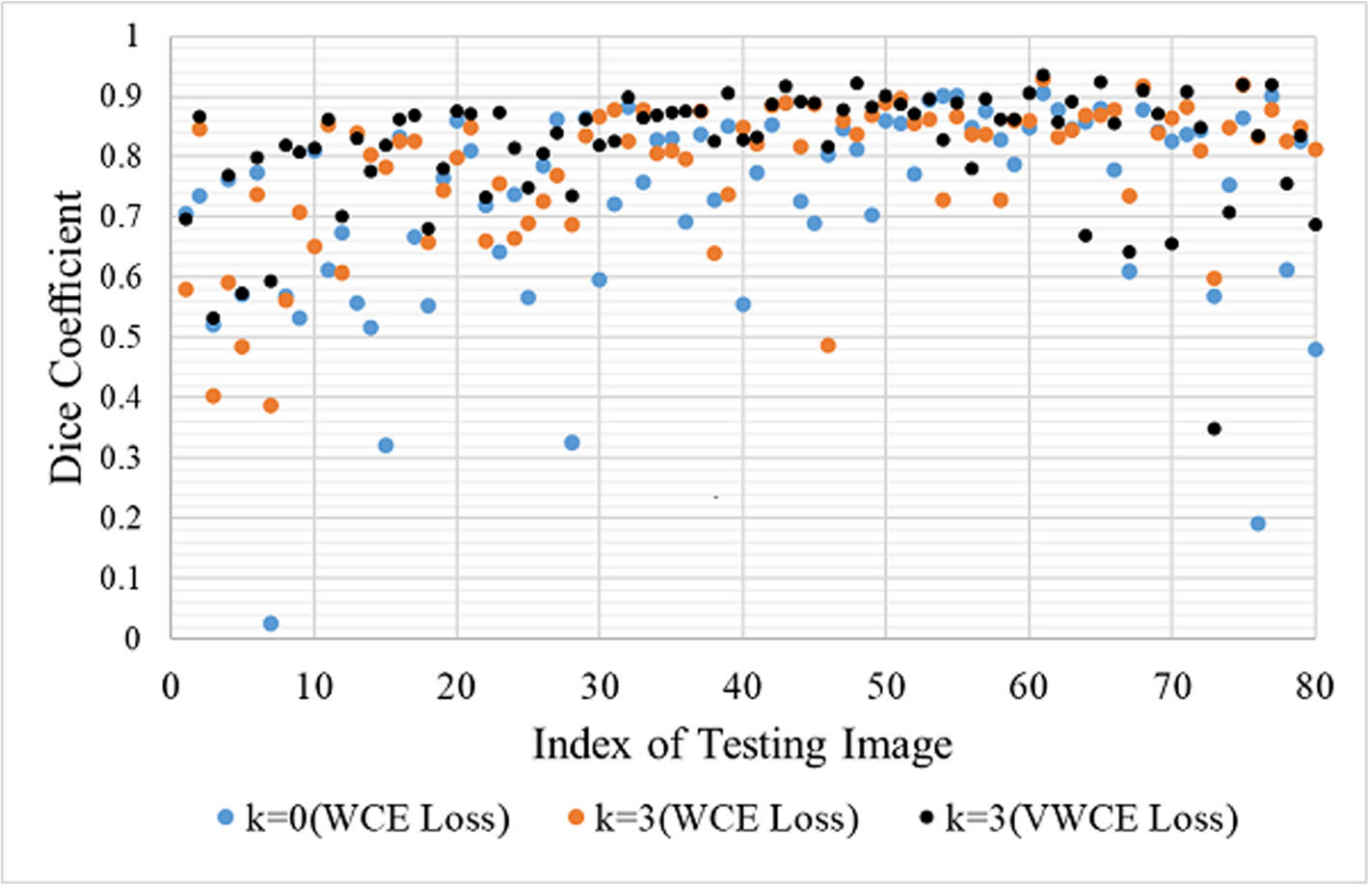
DSC of each case in the testing dataset with different parameters. The index of images is ranked according to the volume of renal tumors

The DSC of each case in the testing dataset with different parameters is shown in Fig. 8. For testing dataset, it can be seen that our three-stage training strategy with VWCE loss has significantly improved the segmentation results in most images and achieves the best improvement of DSC.

### Comparison with other methods

Three methods including two weakly-supervised methods (SDI and constrained-CNN) and one fully-supervised method (UNet) were used to compare with our proposed method. These methods are briefly summarized in Existing methods section. For model training, the computation time of our proposed method is about 48 h, the SDI method is about 80 h, and the constrained-CNN and fully-supervised UNet are about 24 h. for model testing, the computation time of our proposed method is similar to the fully-supervised method. Our network can generate the segmentation result of a single image in a few seconds

Table 3 is the comparison of segmentation results among our method, the other two existing weakly-supervised methods and fully-supervised method. We only compared the bounding box with *d* = 5 for simplicity. Experiments show that our method achieves the best results of DSC, HD and ASD, which are 0.826, 15.811 and 2.838 respectively. In terms of DSC, neither SDI nor Constrained-CNN reaches the values higher than 0.8. One thing worth to be mentioned is that the evaluation metrics are not improved effectively in SDI after MC since we deal with it in 2D situation. When the margin is lower than 5, the performance of our method is close to the results obtained by the fully-supervised UNet.

**Table 3:** Comparison of testing results with different methods

	DSC	HD	ASD
Constrained-CNN [21]	0.705	102.178	8.271
Constrained-CNN [21] + 3D MC	0.712	20.939	5.493
SDI [5]	0.766	73.514	4.639
SDI [5] + 2D MC	0.766	72.368	4.524
Ours (*d* = 5)	0.820	37.633	3.879
Ours (*d* = 5) + 3D MC	**0.826**	**15.811**	**2.838**
UNet [32] (Fully-supervised)	0.849	84.69	4.886
UNet [32] (Fully-supervised) + 3D MC	0.859	14.252	2.048

**Fig. 9:**
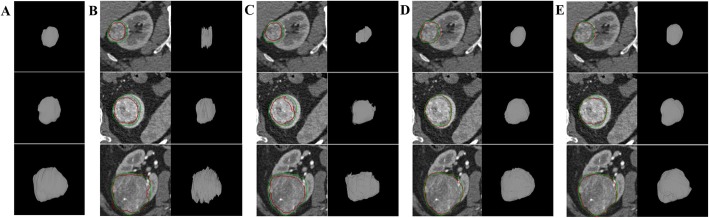
The comparison of the results from three testing images obtained by different methods: 3D ground truth (**a**), SDI (**b**), Constrained-CNN(**c**), the proposed method (**d**) and fully-supervised method (**e**). Contours in green and red correspond to ground truth and segmentation results respectively

Figure 9 shows the comparison of segmentation results obtained by different methods. For SDI method, the shape of the segmented renal tumor in 3D is not continuous as shown in Fig. 9(b). Furthermore, SDI and Constrained-CNN still suffer from the under-segmentation problem. While, our proposed method (d) presents better segmentation results which are similar to the fully-supervised method (e) in visual.

## Discussion

According to our experimental results, our proposed weakly-supervised method can provide accurate renal tumor segmentation. The major difficulty for weakly-supervised methods is that feature maps learned by CNN models can be misled by under- or over-segmentation in the weak masks. Therefore, the key factor in weakly-supervised segmentation is to generate reliable masks from the input weak labels. In this paper, the application of pseudo masks generation and group training improve the quality of the weak masks used for the final CNN model training as shown in Tables 1 and 2.

Furthermore, as shown in Fig. 8, the DSCs of large and small tumors are relatively low. It is easy to understand that the DSCs of the small renal tumors are sensitive to the over- or under-segmentation in the predictions. While in large tumor, the shape and texture of the tumor are complicated, which leads to the difficulties of the segmentation. Although this problem exists in all three methods, our proposed method shows the most significant improvement compared with the other two methods.

Finally, one limitation of this study is the lack of validation of the final CNN model with external datasets. The training and testing datasets in this paper are from the same hospital. Additional validation of the final CNN model with multi-center or multi-vendor images will be performed in the future. Due to the differences in image acquisition protocols or the other factors, the CNN model trained in this paper may not be able to achieve a similar performance on the other datasets. However, the parameters in our model can be optimized by fine-tuning with the external datasets to improve the accuracy. In particular, the main advantage of our method is the use of weak labels for network training, which does not take much time for radiologists to generate bounding-box labels.

## Conclusion

In this paper we have presented a novel three-stage training method for weakly supervised CNN to obtain precise renal tumor segmentation. The proposed method mainly relies on the group training and weighted training phases to improve not only the efficiency of training but also the accuracy of segmentation. Experimental results with 200 patient images show that the DSCs between ground truth and segmentation results can reach 0.834, 0.826 when the margin of bounding box was set to 0 and 5, which are close to the fully-supervised model which is 0.859. The comparison between our proposed method and the other two existing methods also demonstrate that our method can generate a more accurate segmentation of renal tumors than the other two methods.

## Data Availability

The clinical data and materials used in this paper are not open to public, but are available from the corresponding author on reasonable request.

## Abbreviations

ASD: Average surface distance
CE: Cross-entropy
CNN: Convolutional neural network
ConvCRFs: Convolutional conditional random fields
CRF: Conditional random field
CT: Computed tomography
CTA: Computed tomographic angiography
DSC: Dice coefficient
FCN: Fully convolutional network
HD: Hausdorff distance
LPN: Laparoscopic partial nephrectomy
MAP: Maximum a posteriori
MC: Maximum connected component
MR: Magnetic resonance
RCC: Renal cell carcinoma
ROI: Region of interest
SVM: Support vector machine
VWCE: Voxel-wise weighted cross-entropy
WCE: Weighted cross-entropy
